# A Low-Cost, High-Performance System for Fluorescence Lateral Flow Assays

**DOI:** 10.3390/bios3040360

**Published:** 2013-10-21

**Authors:** Linda G. Lee, Eric S. Nordman, Martin D. Johnson, Mark F. Oldham

**Affiliations:** Song Diagnostic Research LLC, 1 Megans Lane, Woodside, CA 94062, USA; E-Mails: eric@songdx.net (E.S.N.); marty@songdx.net (M.D.J.); mark@songdx.net (M.F.O.)

**Keywords:** fluorescence lateral flow immunoassay, phycoerythrin, biotinylated BSA, hCG

## Abstract

We demonstrate a fluorescence lateral flow system that has excellent sensitivity and wide dynamic range. The illumination system utilizes an LED, plastic lenses and plastic and colored glass filters for the excitation and emission light. Images are collected on an iPhone 4. Several fluorescent dyes with long Stokes shifts were evaluated for their signal and nonspecific binding in lateral flow. A wide range of values for the ratio of signal to nonspecific binding was found, from 50 for R-phycoerythrin (R-PE) to 0.15 for Brilliant Violet 605. The long Stokes shift of R-PE allowed the use of inexpensive plastic filters rather than costly interference filters to block the LED light. Fluorescence detection with R-PE and absorbance detection with colloidal gold were directly compared in lateral flow using biotinylated bovine serum albumen (BSA) as the analyte. Fluorescence provided linear data over a range of 0.4–4,000 ng/mL with a 1,000-fold signal change while colloidal gold provided non-linear data over a range of 16–4,000 ng/mL with a 10-fold signal change. A comparison using human chorionic gonadotropin (hCG) as the analyte showed a similar advantage in the fluorescent system. We believe our inexpensive yet high-performance platform will be useful for providing quantitative and sensitive detection in a point-of-care setting.

## 1. Introduction

**Figure 1:**
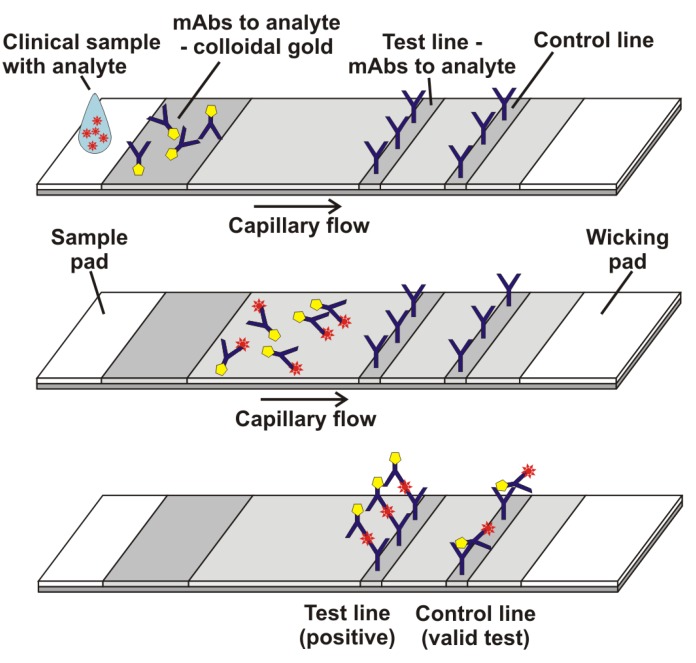
Schematic of a lateral flow assay with colloidal gold as label.

Lateral flow technology [1] is used for the detection of proteins, viral antigens and small molecules and enables rapid point-of-care diagnostics of infectious diseases (malaria [2], dengue [3,4], and HIV [5]) as well as cardiac markers (troponin [6]) and cancer biomarkers (prostate specific antigen [7]). The format utilizes a sandwich immunoassay: two antibodies are ultimately bound to an analyte in a sandwich fashion. One antibody (mAb) is initially bound non-covalently in a horizontal stripe on a narrow strip of nitrocellulose. The nitrocellulose is blocked with protein to prevent nonspecific adherence of analyte and other proteins, and the analyte and a second, labeled antibody (typically labeled with colloidal gold) is allowed to flow up the nitrocellulose. A “sandwich” of the analyte and the two antibodies forms on the stripe and appears as a visible, reddish line. Typically, an absorbent pad containing the gold-labeled antibody is used to deliver the reagent, and a control line containing antibody to the Fc portion of the gold-labeled antibody is located upstream of the test line. A diagram of the process is illustrated in Figure 1. 

The most common reporter entity is colloidal gold. Antibodies can be noncovalently or covalently attached to gold and visual detection of the stripe is simple and robust. Gold is stable under exposure to heat and light, and degradation is limited primarily by the stability of the protein. Disadvantages include a very limited quantitative dynamic range, and a limit of detection far below theoretical expectations [8,9] that is sometimes inadequate even with reader systems. 

Fluorescence is an obvious choice as a method to improve both the limit of detection and the dynamic range in lateral flow. The primary advantage of fluorescence over absorbance systems is the dark and uniform background that is achieved by efficient blocking of the excitation light. Fluorescence detection also provides a wide dynamic range, since the light emitted is proportional to the concentration, while the amount of light reflected after absorption is a nonlinear function of concentration. Generally, fluorescence systems tend to be expensive due to the expensive light sources required to illuminate the fluorescent reporters, the interference filters and detection systems required to process and capture the emitted light, and the data processing required to produce the result. Several reports have described the use of fluorescence in lateral flow systems [10,11,12] but their results do not show a sufficient advantage of using fluorescence instead of gold in either sensitivity or dynamic range that would justify the extra cost and complexity.

We have created an inexpensive reader system using LED light sources and readily available plastic and colored glass filters. The image is captured using the camera on a mobile phone and subsequently downloaded and processed on a computer. We ultimately envision a new phone application that would enable on-phone data processing and reporting, thus providing all computer functions on the mobile device. Here, we use this system to explore the characteristics of various fluorescent reporters in lateral flow systems. We compare the signal and nonspecific binding characteristics of several different fluorescent dyes. We also use one of these dyes, R-phycoerythrin, in two different model systems in a lateral flow format and compare their performance to colloidal gold. 

## 2. Experimental Section

### 2.1. Materials

Biotinylated BSA and streptavidin were purchased from Thermo Fisher Scientific (Rockford, IL, USA). R-PE streptavidin and Alexa Fluor 532 streptavidin were purchased from Life Technologies (Carlsbad, CA, USA). BSA was purchased from Sigma-Aldrich (St. Louis, MO, USA). Brilliant Violet 605 streptavidin was purchased from BioLegend (San Diego, CA, USA). Chromeo 494 streptavidin was purchased from Active Motif (Carlsbad, CA, USA). Atto 465 streptavidin and Atto 430-LS streptavidin were purchased from Atto-tec (Siegen, Germany). Gold-labeled streptavidin was purchased from Innova Biosciences (Cambridge, UK). Biotin-X-NHS ester was purchased from AAT Bioquest (Sunnyvale, CA, USA). Goat polyclonal anti-hCG, beta hCG, and mouse monoclonal anti-hCG were purchased from Scripps Laboratories (San Diego, CA, USA). Lateral flow materials (glass fiber, cellulose, nitrocellulose) were manufactured by Millipore Corporation (Bedford, MA, USA) and GE Healthcare (Buckinghamshire, UK), Backing material was obtained as a sample from DCN Diagnostics (Carlsbad, CA, USA). 

Colored glass optical filters were purchased from Thor Labs (Newton, NJ, USA). Interference filters were purchased from Chroma (Bellows Falls, VT, USA). Plastic filters were purchased as a booklet from Edmund Optics (Barrington, NJ, USA). The LEDs (Phillips Luxeon^®^ Star) and LED optics (except 405 nm LED) were purchased from Quadica Developments Inc (Brantford, ON, Canada). The 405 nm LED and reflector was purchased from Super Bright LEDs (Saint Louis, MO, USA). An iPhone 4 was purchased from Apple Computer (Cupertino, CA, USA). ProCamera was purchased from Cocologics (Mannheim, Germany) through the Apple App store. ImageJ software was downloaded from the NIH website (National Institutes of Health, Bethesda, MA, USA).

### 2.2. Optics Breadboard Design and Construction

The following description of the breadboard is specific for analysis of R-phycoerythrin (R-PE). 

**Figure 2:**
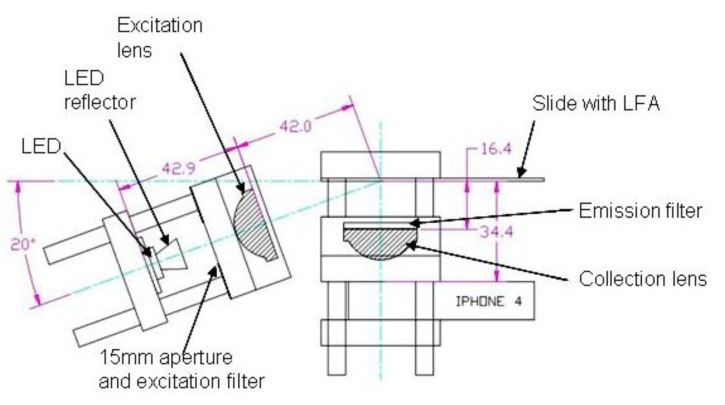
Layout of optics breadboard.

To facilitate easy setup modification and allow use of 1″ optics and filters the optics breadboard (BB) was constructed using 30 mm cage components (Thorlabs, Newton, NJ, USA). The cage components were secured to an aluminum plate positioning optics as shown in Figure 1, allowing motion of one plate to clamp the smartphone. The excitation source was a 505 nm LED providing 122 lm at 700 mA (SR-01-E0070, Quandrica Developments, Brantford, ON, Canada). The LED current was controlled by a 700 mA externally dimmable DC driver (A011-D-V-700, LEDdynamics Quandrica Developments) powered by eight AA batteries with holder (Mouser Electronics). A 20 KΩ potentiometer (652-3386P-1-203LF, Mouser Electronics) was used to control the LED current (normally set to full (700 mA) except when setting exposure). A power switch (611-CA22J72207PQ, Mouser Electronics) was provided to prevent draining of the batteries when not in use. The LED was mounted to the cage support endplate using precut thermal adhesive tape (LXT-S-12, Quandrica Developments) with a 7°, 11 mm reflector (Dialight). The excitation filter was provided by two 0.003″ thick plastic films (Supergel #69 brilliant blue, Rosco). The excitation beam was focused using a 25 mm diameter, 25 mm FL acrylic lens (NT48-170, Edmund Optics). A schematic of the optics breadboard for analysis of the lateral flow assay (LFA) and dot blots is shown in Figure 2.

The scattered emission light was first filtered using a single 0.003″ thick plastic film (Supergel #15 Deep Straw, Rosco) along with a 2 mm thick, Schott OG570 colored glass filter (FGL570, Thorlabs). The emission light was semi-collimated using a 25 mm diameter, 25 mm FL acrylic lens (NT48-170, Edmund Optics) for collection using the smartphone (iPhone 4). Apertures were hand cut out of black plastic and the system was shielded from room light using a hand fabricated black foam core box.

Image capture was done using the ProCamera app with the following settings: Lightbox off, expert mode on, self timer 5 s. The exposure time (varied) and ISO settings (always set to ISO 80) were set by trial and error on a selection of points on the image from a fluorescent target (paper marked with orange highlighter) and locked. Once the camera settings were locked the potentiometer was set for max current (700 mA) and images were taken of nitrocellulose mounted to standard 1″ × 3″ glass slides that were temporarily secured using double sticky tape to the cage endplate. 

Several variations of the optics breadboard, shown in Table 1, were used to analyze different fluorescent dyes. 

**Table 1:** Breadboard variations: dyes, LEDs and filters.

Dye	LED	Excitation Filter	Emission Filter
Atto 430-LS	447 nm, 910 mW at 700 mA, Luxeon^®^ Star with 7° reflector	2 mm thick Schott BG3 colored glass	Chroma 565/40 M interference filter after lens
Atto 465	447 nm, 910 mW at 700 mA, Luxeon^®^ Star with Dialight 7° reflector	2 mm thick Schott BG3 colored glass	Chroma 520/30 M interference filter after lens
Brilliant Violet 605	405 nm, 475 mW at 700 mA, Prolight with 10° Prolight reflector	2.5 mm thick Hoya B-390 colored glass	2 mm Schott OG570 colored glass before lens
Chromeo 494	505 nm, 122 lm at 700 mA, Luxeon^®^ Star with Dialight 7° reflector	Chroma 520/30M interference filter after lens	Two 2 mm Schott OG570 colored glass filters after lens
Alexa Fluor 532	530 nm, 150lm at 700 mA, Luxeon^®^ Star with Dialight 7° reflector	Chroma 520/30M interference filter after lens	Two 2 mm Schott OG570 colored glass filters after lens
R-Phycoerythrin	505 nm, 122lm at 700 mA, Luxeon^®^ Star with Dialight 7° reflector	Two 0.003″ thick plastic films (Supergel #69, brilliant blue)	One 0.003″ plastic film (Supergel #15 Deep Straw) and one 2 mm Schott OG570 colored glass filter before lens

### 2.3. Image Analysis

Captured images were analyzed using ImageJ software. Images were cropped and rotated so the flow direction was horizontal. The images were converted to RGB format and the appropriate color selected (red for R-PE, green for colloidal gold). A freehand line was drawn around the fluorescent zone and the intensity, area, min and max were collected using the measure icon. A rectangle was drawn and used for a plot profile across the illuminated area. Column averages to the left and right of the spot were used to find a baseline for the data. If different exposure times were used the signals were appropriately scaled. The total signal over baseline was calculated and then plotted on log-log scales with a power fit trend line using Microsoft Excel 2003. For colloidal gold the same method was used, except the total signal below the baseline (absorbance) was used.

### 2.4. Nonspecific Binding Measurement

Dye-labeled streptavidin was diluted to create a two-fold dilution series in phosphate buffered saline (PBS) in the range of 0.63–40 µg/mL. To generate the spots for the signal data, the dilution series was spotted (1 µL) on untreated nitrocellulose dried and mounted onto glass slides. To generate strips for the nonspecific binding data, strips of nitrocellulose (5 mm × 20 mm) were initially immersed into 5% bovine serum albumin in PBS for 30 min, rinsed and dried. The strips were then immersed into 0.5 mL of the dilution series for 20 min, rinsed in 1× PBS, dried and mounted on glass slides. Images of the signal (spots) and of the nonspecific binding (strips) were collected as described above. 

### 2.5. Lateral Flow with Streptavidin, Biotinylated BSA, and Either R-PE-Streptavidin or Gold-Streptavidin

**Figure 3:**
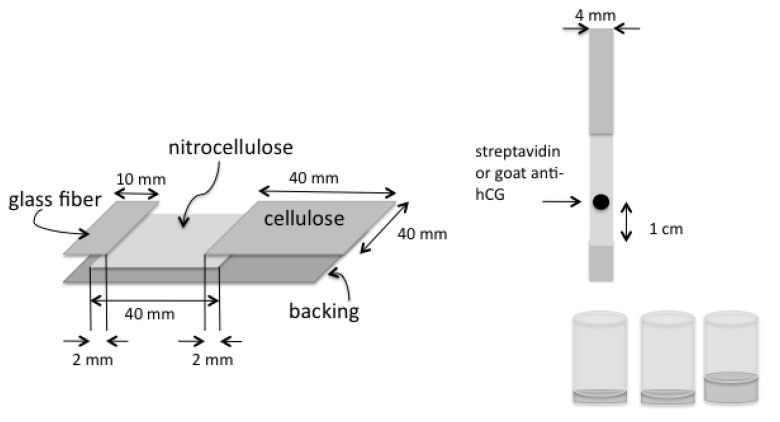
Construction and use of lateral flow strips.

Nitrocellulose (Millipore HiFlow Plus HFB13502) was cut (4 cm × 4 cm) and mounted onto an adhesive backing 8 mm from the edge. Glass fiber pad (Millipore GFCP20300) was cut into a rectangle (10 mm × 4 cm) and mounted on the edge of the backing, overlapping the nitrocellulose by 2 mm. Cellulose (GE Healthcare, CF3) was cut (4 × 4 cm) and mounted on the backing, overlapping the nitrocellulose by 2 mm. The assembly was cut into 4 mm wide strips. Streptavidin was spotted at 4 mg/mL in 0.5 µL aliquots 1 cm above the absorbent pad. A four-fold dilution series of biotinylated BSA in 1% BSA/PBS was prepared, in concentrations ranging from 63 pg/mL to 16 µg/mL. The strips were dipped successively into 20 µL of each concentration of the dilution series, followed by 20 µL of R-PE streptavidin (0.01 mg/mL in 1% BSA/PBS), and then 50 uL 1% BSA/PBS. The solutions were contained in 2.0 mL cylindrical collection tubes (Affymetrix). At each step the liquid was allowed to totally absorb onto the strip before immersion into the next solution. The strips were air-dried and mounted on glass slides. A schematic of the strip construction and use is shown in Figure 3.

### 2.6. Lateral Flow with Anti-hCG, β-hCG, and Either Biotinylated Anti-hCG + R-PE-Streptavidin or Biotinylated Anti-hCG + Gold-Streptavidin

Strips were constructed as described above. Mouse monoclonal anti-hCG was biotinylated with biotin-X-NHS at pH 9.2 and excess reagent removed on a Sephadex G-25 column. Goat polyclonal anti-hCG was spotted at 4 mg/mL in 0.5 µL aliquots 1 cm above the absorbent pad. A four-fold dilution series of hCG in 1% BSA/PBS was prepared, in concentrations ranging from 1,000 ng/mL to 63 pg/mL. The strips were dipped successively into 20 µL of each concentration of the dilution series, 20 µL of a mixture containing 0.01 mg/mL R-PE streptavidin and 0.005 mg/mL biotinylated mouse monoclonal anti-hCG in 1% BSA/PBS, and 50 µL of 1% BSA in PBS. The strips were air-dried and mounted on glass.

## 3. Results and Discussion

### 3.1. Survey of Fluorescent Reporters; Ratio of Signal to Nonspecific Binding

Many fluorescent entities are available commercially, and conveniently, many are available as streptavidin conjugates. Fluorescent compounds can be divided into two types, soluble “small molecules” and particles, such as fluorescent latex beads, quantum dots or europium chelates. We have focused on the soluble type of fluorescence molecules, and in particular, fluorescent dyes with long Stokes shifts (the difference between the excitation and emission maxima) ranging from 55–200 nm. For comparison, we used Alexa Fluor 532, a conventional dye with a Stokes shift of 22 nm. We initially began our survey with dot blots using biotinylated BSA spotted on nitrocellulose and detection of bound dye-labeled streptavidin. However, it soon became clear that the background fluorescence was limiting the sensitivity for several of the dyes, leading us to search for a quantitative approach to characterize the nonspecific binding of each of the dyes to blocked nitrocellulose. 

Our quantification method to allow comparison of the nonspecific binding characteristics of various dyes relied on determining the ratio of the signal to the nonspecific binding (NSB) signal for each dye conjugated to streptavidin. The signal from spotting a fixed volume (1 µL) of a dilution series of a dye-labeled streptavidin and the signal from dipping pre-blocked nitrocellulose in the same dilution series were plotted. Linear fits to the data were calculated using Excel, and the ratio of the two slopes gave a unitless number, the ratio of signal to NSB. This number is independent of the sensitivity of detection of each system. This system independence is necessary since the various dyes require different LEDs and filters. 

**Figure 4:**
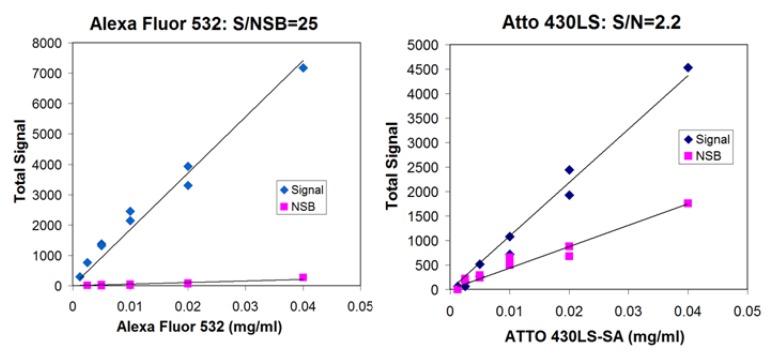
Alexa Fluor 532 has a good ratio of signal to nonspecific binding (S/NSB) compared to Atto 430LS. Each fluorophore is conjugated to streptavidin, spotted on nitrocellulose and the signal read in the breadboard (signal, blue diamonds). Strips of nitrocellulose that have been blocked with BSA are immersed in each solution and read in the breadboard (nonspecific binding, magenta squares). The ratios of the two slopes are reported as the S/NSB ratio.

Shown in Figure 4 are the signal and NSB data for two of the dyes, Alexa Fluor 532 and Atto 430LS. Table 2 shows the ratio of signal to NSB for all the dyes that we analyzed. Surprisingly, even though all the dyes were very water-soluble, they showed a wide range in ratios of signal to NSB. Brilliant Violet 605 streptavidin was extraordinarily “sticky”, actually producing greater signal in the nonspecific binding mode than the signal mode for each dilution of dye-labeled streptavidin. Alexa Fluor 532 streptavidin and R-PE streptavidin were the least sticky. It is clear from these results that in addition to the inherent brightness of a fluorescent dye, the ratio of signal to NSB is a key characteristic in determining the utility of a dye in lateral flow. A dye with a high ratio of signal to NSB will have a good dynamic range, since high concentrations of dye can be used to saturate high concentrations of analyte without causing too much background for low concentrations of analyte.

**Table 2:** Signal to nonspecific binding ratio for fluorescent dyes conjugated to streptavidin.

Dye-labeled streptavidin	Signal/NSB
R-PE	50
Alexa Fluor 532	27
Atto 430-LS	2.2
Atto 465	1.6
Chromeo 494	1.4
Brilliant Violet 605	0.15

### 3.2. Fluorescence Reader System

We envisioned a fluorescence lateral flow system of strip and reader that is both low-cost and high-performance. To achieve this, our first goal was to find a fluorescent reporter that had a long Stokes shift; that is, where the excitation maximum is well separated from the emission maximum. If the Stokes shift is greater than approximately 70 nm, extremely low-cost colored plastic or colored glass can replace costly interference filters that are typically used in fluorescence readers. For the light source, different LEDs with a variety of wavelengths were used. Instead of a scanning system to detect the signal, we used the camera in a common smartphone, the iPhone 4. This allowed variation in the length of exposure, extending the dynamic range of the assay. Ultimately, data analysis could be done on the mobile device after the development of a mobile image analysis application. For data shown here, we downloaded the images to a computer and used ImageJ for the analysis. 

**Figure 5:**
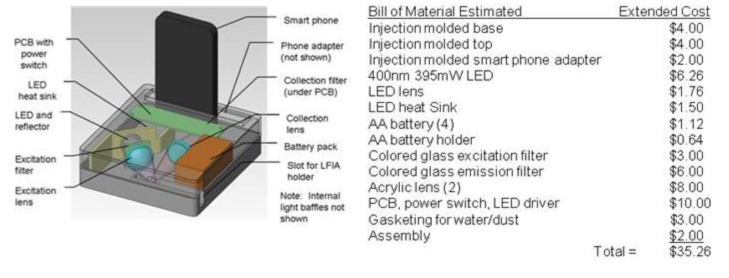
Fluorescence system concept and estimated cost. Functions include LFIA detection, analysis and communications. Cost of smartphone not included.

Our ultimate goal is to create a manufacturable reader system that would be an accessory to a smartphone. Shown in Figure 5 is an early concept of such a reader; we have estimated the final cost based on the component parts. 

### 3.3. Lateral Flow with a Sandwich of Streptavidin, Biotinylated BSA, and Labeled Streptavidin

Following the method of Juntunen *et al*. [13], we tested various streptavidin conjugates using a simplified lateral flow format. The pad containing the labeled reagent was omitted; instead, a simplified lateral flow strip consisting of glass fiber feeding pad, nitrocellulose and cellulose absorption pad on a backing was constructed. A spot rather than a stripe of reagent was applied to the nitrocellulose. The strip was dipped into three successive solutions of analyte, labeled reagent, and then buffer. Each of these solution contained 1% BSA to prevent nonspecific adhesion of the proteins to the nitrocellulose. The strips were then allowed to dry, and read on the breadboard. This format was used to compare fluorescence (R-PE) and absorbance (gold) assays, in which all components were identical, excepting the labeled streptavidin.

**Figure 6:**
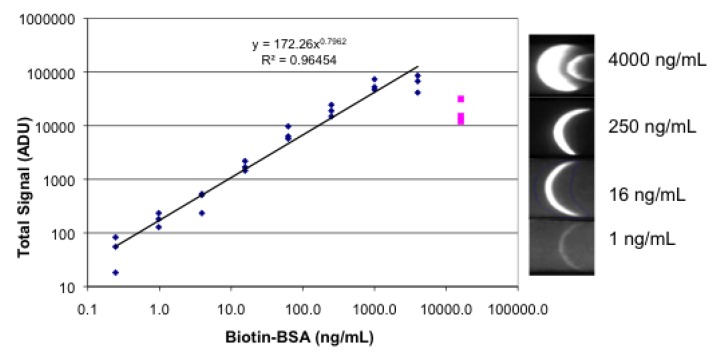
Fluorescence lateral flow images and plot. A spot rather than the conventional stripe of streptavidin was applied to the nitrocellulose. The spot diameter was approximately 3 mm. Dilutions of biotinylated BSA, followed by R-PE streptavidin, followed by buffer were absorbed onto the strips. Each concentration was tested in triplicate. Images were obtained in a breadboard equipped with an iPhone 4 and ProCamera app; a sample of the images is shown on the right. Image analysis was done with Image J and the results plotted.

Figure 6 shows the results of a fluorescence lateral flow assay that utilized a sandwich system of streptavidin, biotinylated BSA, and R-PE-labeled streptavidin. Streptavidin was spotted down on the strips and allowed to dry. A four-fold dilution series of biotinylated BSA in 1% BSA was prepared. Each strip was dipped successively into 20 µL of the dilution series, then into 20 µL of R-PE streptavidin, and finally into 50 µL 1% BSA. After drying the strips were imaged. The images are a crescent shape rather than a filled-in spot, showing that the streptavidin-biotin-RPE-streptavidin sandwich is formed at the leading edge of the spot, or as soon as the eluting reagents enter the “test” zone.

The results show a very wide dynamic range in both analyte concentration (0.4–4,000 ng/mL) and signal (1,000-fold difference). At the upper end of the concentration range (16,000 ng/mL), the signal is no longer linear and the data was not included in the fit. The loss of linearity is due to the “prozone effect” that occurs when the concentration of analyte is high enough to saturate both antibodies, precluding the formation of the antibody-analyte-antibody sandwich [14]. 

**Figure 7:**
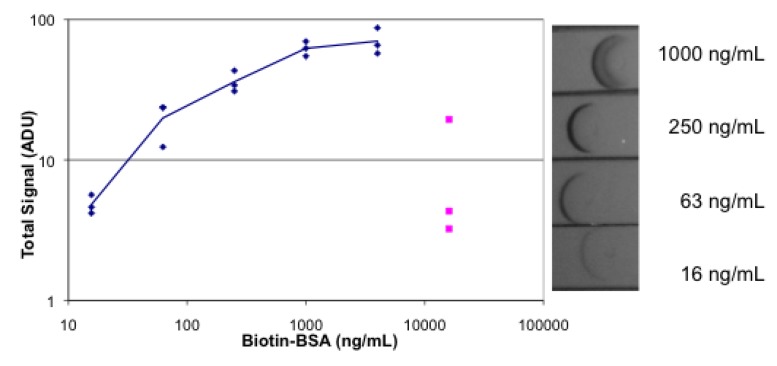
Absorbance lateral flow images and plot. The strips were spotted with streptavidin. Dilutions of biotinylated BSA, followed by gold-labeled streptavidin, followed by buffer were absorbed on the strips. Each concentration was tested in triplicate. Images were obtained with the camera of an iPhone 4. Image analysis was done with Image J and the results plotted. A sample of the images is shown on the right.

Figure 7 shows the analogous assay with the substitution of colloidal gold for the R-PE on streptavidin and flash photography instead of fluorescence detection. Compared to the fluorescence assay, the absorbance assay has a narrower useful concentration range as well as a less sensitive limit of detection. The absorbance data has a dynamic range of 16–4,000 ng/mL of biotinylated BSA; the signal is not a linear function of concentration. The dynamic range of the signal is also smaller; the difference between the highest and the lowest signal is only 10-fold. The prozone effect is observed at 16,000 ng/mL as much reduced signal.

### 3.4. Lateral Flow Using a Sandwich of Polyclonal Anti-hCG, hCG, and Biotinylated Monoclonal Anti hCG/Labeled Streptavidin

**Figure 8:**
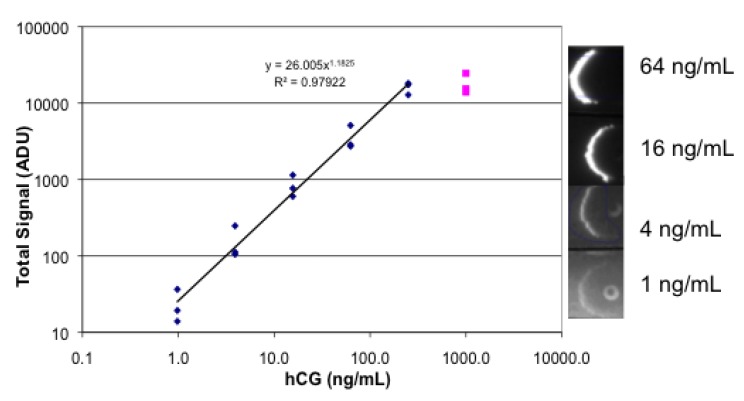
Images and data for fluorescence lateral flow analysis of hCG. Lateral flow strips were spotted with goat anti-hCG, then dipped successively in a dilution series of hCG, followed by RPE streptavidin mixed with biotinylated mouse anti-hCG, followed by buffer. Each concentration of hCG was tested in triplicate.

**Figure 9:**
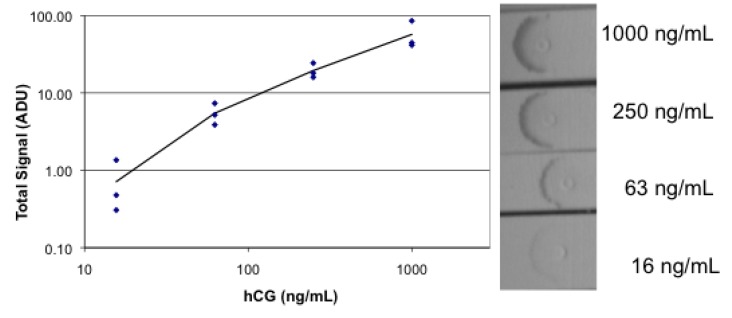
Images and plot of absorbance lateral flow analysis of hCG. Lateral flow strips were spotted with goat anti-hCG, then dipped successively in a dilution series of hCG, followed by gold streptavidin mixed with biotinylated mouse anti-hCG, followed by buffer. Each concentration of hCG was tested in triplicate.

Analysis of human chorionic gonadotropin (hCG) was also performed with the simplified lateral flow system with both fluorescence and absorbance measurement. The sandwich system for fluorescence consisted of polyclonal goat anti hCG spotted on the strip, anti hCG as the analyte, and biotinylated mouse monoclonal anti hCG mixed with R-PE streptavidin. The results of testing strips in a four-fold dilution series are shown in Figure 8. The prozone effect is evident at 1,000 ng/mL with a non-linear data point. Evidence of the pipette tip used for spotting the goat antibody appears as a fluorescent spot, perhaps due to a high local concentration of antibody. The titration with absorbance measurement using biotinylated mouse monoclonal anti-hCG mixed with gold-labeled streptavidin is shown in Figure 9. As before, the fluorescence measurement shows both greater sensitivity and dynamic range than the absorbance measurement.

### 3.5. Photobleaching of Alexa Fluor 532 and R-PE

**Figure 10:**
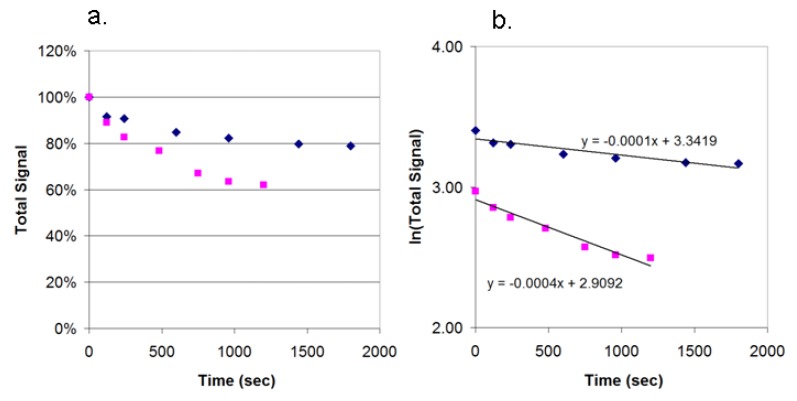
Photobleaching of R-PE streptavidin (pink) and Alexa Fluor 532 streptavidin (blue). The compounds were spotted on nitrocellulose and exposed to constant illumination with a 505 nm LED. Images were collected at time intervals. (**a**) Data was normalized to the initial values; (**b**) A plot of the natural logarithm of the signal. Half-lives were determined by the equation t_1/2_ = ln2/k, where k is the negative slope of the natural log plot.

R-PE is reported to be less photostable than organic dyes. We tested the stability in our breadboard by illuminating spots of R-PE streptavidin and spots of Alexa Fluor streptavidin and recording the loss of signal over time. Figure 10 shows plots of signal *vs.* time for both dyes as a percentage of the initial signal and the natural logarithm of the signal. Under constant LED illumination, Alexa Fluor 532 is more stable (t_½_ = 7,000 s) than R-PE (t_½_ = 2,000 s). Both are expected to be sufficiently stable under normal storage conditions of lateral flow strips.

## 4. Conclusions

We have evaluated several soluble fluorescent dyes conjugated to streptavidin and determined a method to evaluate the signal *vs.* nonspecific binding characteristics of these compounds on lateral flow materials. The method is simple and should be readily applicable to fluorescent particles, such as latex beads and quantum dots, as these are also readily available conjugated to streptavidin. We have built an illumination device coupled with a smartphone to allow detection and analysis of R-PE in lateral flow format for various fluorescent dyes. We show superior performance of the fluorescence systems compared to colloidal gold in lateral flow analyses of biotinylated BSA and hCG in both sensitivity and dynamic ranges of analyte concentration and signal level. Further improvement can likely be achieved by machine deposition of antibody on the nitrocellulose in stripes rather than by manual spotting, direct attachment of R-PE to the antibody rather than via streptavidin/biotin, and further reduction in nonspecific binding by evaluation of different materials, buffers and blocking agents. A custom smartphone application can allow longer exposure times (currently limited to 1/15 s by the ProCamera application). Additional improvements are likely using image processing techniques such as flat field correction and combining of multiple images. Testing of a lateral flow strip complete with a conjugate pad to hold and deliver fluorescent antibody will be necessary to determine reagent stability and performance. 

There are both advantages and disadvantages to using fluorescence in lateral flow assays. The advantages include higher sensitivity, and wider dynamic ranges in analyte concentration and in signal level compared to absorbance measurements. The disadvantages include the requirement of a reader, since the fluorescent signals are only visible to the eye at a high concentration. In addition, the chemistry of conjugation of fluorescent materials requires single or multistep covalent conjugation chemistry. Attachment of antibodies to colloidal gold, by contrast, is often achieved by pH dependent passive absorption.

Smartphones have been used as sensitive detectors in biological analysis for applications ranging from non-fluorescent lateral flow readers [15], to albumen testing [16] and imaging cells in a portable flow cytometer [17]. The advantages of using smartphones as an integral part of the instrument include readily upgradable applications, the ability to facilitate the the automation of disease tracking, the compact size and ubiquity of smartphones, and inherent obviation of the need for a bulky computer. Disadvantages include the inherent difficulty in obtaining FDA/CE approval due to the constantly changing standards. These changing standards also result in the requirement for a very flexible interface in the illumination device.

In summary, although existing gold lateral flow strips are robust, simple and good for positive or negative determination, use of fluorescence offers the advantages of quantitation and increased sensitivity when these requirements are needed. Examples of where these requirements may offer significant advantage include accurate quantitation of cardiac markers [18]; quantitation and identification of environmental contaminants [19]; detection of low parasite levels prior to malaria recrudescence [20]; and quantitation of tumor markers such as prostate specific antigen and CA-125 [21]. We have demonstrated a route to offer these advantages in a point-of-use setting.
